# Ophthalmology

**Published:** 1897-03

**Authors:** F. R. Cross


### OPHTHALMOLOGY.

**Embolism of the central artery of the retina,** probably in a majority of the cases, affects the main trunk of the vessel and is accompanied by complete loss of sight. A large number of cases, however, are on record where only one or more branches of the main artery are plugged. The sudden onset of the lesion may cause complete loss of sight for a time, but the only part of the retina which is permanently disabled is that which depends on the occluded branches for its blood-supply.

A paper of Wagenmann of Jena[3] describes the pathological examination of an eyeball, which was removed at the death of the patient a year after complete monocular amaurosis had

[3] *Arch. f. Ophth.*, 1894, xl. 221; abstract in *Ophth. Rev.*, 1894, xiii. 359.

occurred from embolism of the main central artery. The embolus lay just behind the lamina cribrosa; the central artery was much distended where the plug was situated, but above and below it the lumen of the artery was narrowed. The plug was partially organised, partially degenerating. Circulation might possibly have become re-established by shrinking of the plug or by a paralytic expansion of the vessel. Ascending atrophy of the optic nerve was almost complete; the optic disc was atrophic; the inner layers of the retina were quite atrophic and reduced, but the outer layers (external nuclear and rods and cones) were well preserved.

The main central artery usually bifurcates into a superior and an inferior division, each of which splits into a temporal and a nasal branch. The macula has, however, frequently a special branch coming from the main vessel before its bifurcation, or else coming from the superior temporal artery, or less often from the inferior temporal; while not infrequently there are said to be one or more cilio-retinal vessels which appear at the margin of the disc, and are believed to arise directly from the posterior ciliary vessels and to be free of the arteria centralis retinæ.

Ward Holden[1] gives notes of five cases where embolism of a branch of the retinal artery caused partial damage to the field of vision. (1) A man aged 30. Superior hemianopsia: $V = \frac{6}{6}$. Contraction of inferior branch of central artery. Pallor of lower two-thirds of disc. Cardiac valvular disease. (2) A man aged 23. Superior hemianopsia: $V = \frac{6}{6}$. Contraction of inferior branch of central artery. Middle third of disc pale, lower third grey and atrophied, upper third normal. No heart-murmur, but a history of albuminuria. (3) A woman aged 34. Superior hemianopsia: $V = \frac{6}{6}$, the blind area encroaching on the nasal field at the periphery. Contraction and partial obliteration of the inferior retinal arteries, which probably supplied part of the temporal side of the upper half of the retina. Middle third of disc atrophied, lower third partially vascular from cilio-retinal vessels, upper third normal. Valvular heart disease. (4) A woman aged 33. Scotoma of lower and inner quadrant, implicating the fixation point: $V = \frac{4}{200}$. Embolism in a horizontal branch of superior temporal artery. Later, the temporal side of the disc became atrophic. No cardiac lesion; no albuminuria; but a clear history of syphilis. (5) A man aged 51. General blindness, excepting in a small field corresponding to a small oval area between disc and macula. Retina œdematous; retinal arteries empty or much narrowed, excepting at parts of the periphery and in a limited patch between the disc and the macula, where there is a small artery whose circulation appears to be uninterrupted. The latter case of embolism of the main central artery of the retina, excepting

[1] *Arch. Ophth.*, 1893, xxii.

of a small macular artery, is of special interest. Such a macular artery not infrequently rises from a point of the main vessel before its bifurcation.

Professor Laqueur,[1] of Strasburg, has reported a similar case of embolism of the central artery of the retina, with integrity of the temporal retinal district, and has collected notes of sixteen other cases where the diagnosis was certain as to a similar condition. In Laqueur's case central vision was retained for form and colour. The field was much narrowed from the centre towards the periphery. The retina was œdematous, excepting in a strip running towards the temporal side and including the yellow spot. This strip of normal retina was supplied by an arterial branch on the temporal side, which had a special origin from the arteria centralis retinæ prior to where the embolus had lodged at the usual bifurcation of the vessel.

In each of the sixteen cases quoted by Laqueur there was sudden loss of vision in one eye; later, a partial recovery in the central portion of the visual field, while the periphery continued blind. The normal area of retina lay between the disc and the macula lutea, and usually spread towards the temporal side, sometimes reaching nearly to the periphery, sometimes only just including the yellow spot. The variation in the shape of the unaffected area seemed in all to depend upon the way in which the macular arteries were distributed, and their escape from implication in the general stagnation of the retinal circulation was due to their mode of origin from the main vessels.

A horizontal artery is sometimes seen running close above or below the region of the macula, and sending twigs to supply it from the superior or inferior temporal branches; sometimes a special twig towards the macula runs from the main artery itself, and this may arise after the bifurcation of the vessel, or more centrally than this. This may come from the central artery, or it may pass as a separate vessel through the temporal side of the disc. In the last case, the artery is sometimes looked upon as a cilio-retinal twig coming separately from a posterior ciliary branch; the existence of such a source of supply to the yellow spot region has been used as an explanation for the escape of impairment of function in the central portion when the remainder of the retina is blinded by a plugged central artery. Thus Dr. Wadsworth publishes two cases of retention of central vision with embolism of the retinal artery.[2] The hypothesis, however, that cilio-retinal vessels are concerned with nutrition of the macular portion of the fundus is now opposed by the best authorities, and the cases quoted by Laqueur are all admitted to have depended on supply to the macula from an unobstructed branch of the central artery itself.

[1] *Arch. Ophth.*, 1896, xxv. 525.
[2] *Tr. Am. Ophth. Soc.*, 1896, vii. 585.

The ready way of mapping the area of affected retina, and the examination of the supply and condition of the retinal vessels, may be used as illustrative of the results of embolism of small vessels in the cerebral circulation—where, as in the case of the retina, there is no repair nor recovery by means of a collateral circulation.

Dr. Charles Oliver,[1] of Philadelphia, gives the results of a series of experiments, extending over a long period of time, upon the **action of scopolamine on the iris and ciliary muscle.** The hydrobromate salt appears to be less irritating than that of the hydrochlorate, and at the same time the solution, if prepared with boracic acid, remains for some weeks free of fungoid degeneration. Instillation was made upon the superior limbus of the cornea, the lower lid being everted and the lower canaliculus compressed so as to prevent drainage of the drops into the lachrymal passage. No definite intoxicant effects were observed after repeated droppings, though there occasionally appeared to be giddiness or somnolence. Some stinging or feeling of astringency was noticed in the conjunctiva.

As a mydriatic and cycloplegic for examining the refraction, hydrobromate of scopolamine is most valuable. A single instillation of a drop of the strength gr. j. ad ℥j. gives good dilatation of the pupil in eighteen minutes, the pupil remains fully dilated for from twenty-four to thirty hours, and regains its normal diameter in about seventy hours. One drop of the same strength causes ciliary paralysis in twenty-three minutes, which is maintained for twenty-four to thirty-six hours; the power of accommodation is recovered in four days. One or two instillations of $\frac{1}{480}$ of a grain of scopolamine hydrobromate is an efficient means of estimating the entire ametropia of an eye, and the inconvenience practically lasts for only a couple of days.

Dr. Oliver writes another paper[2] on the **value of the drug in plastic iritis.** A double instillation of $\frac{1}{1000}$ solution was generally used. Its action seems more rapid and active than that of sulphate of atropia four times the strength in cases where the synechiæ are not already well organised. Scopolamine should be preferred in early and incipient cases, but when a prolonged use of a mydriatic is necessary, atropine has advantages, and the two drugs may with benefit be employed alternately: when exacerbations occur, scopolamine appears to be particularly useful in combating the more recent adhesions. Dr. Arthur Hobbs[3] says that a $\frac{1}{2000}$ solution of scopolamine, used four times at intervals of fifteen minutes, will produce a paralysis of the ciliary muscle equal to that following a three days' treat-

[1] *Am. J. M. Sc.*, 1896, cxii. 294. [2] *Ibid.*, 542.
[3] *J. Pract. M.*, 1896, No. 10, p. 428.

ment by sulphate of atropia. Its full effect is much more rapidly reached and the continuance of its action much shorter than is produced by atropine. Hobbs states that scopolamine produces no increase of tension: a statement that greater experience will probably modify.

Many experimenters have arrived at similar conclusions to those of Dr. Oliver. My own experience of the drug verifies its value as a mydriatic and cycloplegic; but in some cases in which I have tried it, it caused dryness of throat, together with definite symptoms of giddiness or even slight mental aberration. The drug, however, seems a perfectly safe one, and I have never experienced serious symptoms such as frequently attended the use of duboisine.

Dr. Oliver Belt[1] gives notes of a serious case of **purulent ophthalmia,** with implication of both corneæ, one of which was said to have perforated, that made a good recovery under **treatment by formalin,** after nitrate of silver and antiseptic treatment had failed. A solution of one part of formalin in 1500 was used for washing the eyes every hour, and the sloughing cornea was daily touched with 1 in 60 formalin solution. After a few days marked improvement was observed, and in a month the case was practically cured. Dr. Belt says: "From the constant progress of the disease until formalin was used, and its rapid subsidence immediately following, I am quite convinced that the eyes would have been lost but for its use." The drug may be specially valuable in arresting corneal ulceration and in lessening corneal opacity.

Dr. Gaetano Vinci[2] gives results of experiments on **eucaine hydrochlorate.** He found a two to five per cent. solution instilled on the eye-surface cause complete local anæsthesia in a few minutes, without dilatation of the pupil and with slight hyperæmia of the conjunctiva instead of the ischæmia caused by cocaine. This vaso-dilator influence may be of advantage in certain cases. The drug, though an excitant, is said to be less poisonous than cocaine, and it is cheaper than the latter drug. Eucaine will probably, however, never displace cocaine in ophthalmic practice; it causes considerable primary irritation, and damages the corneal epithelium. The drug appears to be a slight mydriatic.

F. R. Cross.

[1] *Med. News*, 1896, lxix. 265.

[2] *Arch. f. path. Anat.* [*etc.*], 1896, cxlv. 78; see *Am. J. M. Sc.*, 1896, cxii. 465.

